# Prognostic Value of the Advanced Lung Cancer Inflammation Index in Patients with Lung Cancer: A Meta-Analysis

**DOI:** 10.1155/2019/2513026

**Published:** 2019-07-01

**Authors:** Yi Zhang, Bo Chen

**Affiliations:** ^1^Department of General Surgery, The First People's Hospital of Neijiang, Neijiang, 641000 Sichuan Province, China; ^2^Department of Cardiology, The First People's Hospital of Neijiang, Neijiang, Sichuan Province, China

## Abstract

**Background:**

The advanced lung cancer inflammation index (ALI) has been related to tumor survival in lung cancer (LC) patients. However, these findings regarding the prognostic relevance of ALI in LC were inconsistent. Our study is aimed at characterizing the prognostic significance of low pretreatment ALI in LC cases. Methods. Relevant published studies were systematically searched in several online databases. The combined hazard ratios (HRs) were applied to assess the correlation between ALI and overall/recurrence-free/progression-free survival (OS/PFS/RFS) in LC.

**Results:**

A total of 1587 LC patients from eight articles were recruited. Pooled results indicated that pretreatment ALI was significantly associated with prognosis in cases with LC. Compared to those with high-ALI, LC cases in the low-ALI group had a poorer OS (HR: 1.64, 95% CI: 1.34-1.93, *p* < 0.001). Subgroup analyses further revealed the negative significant prognostic value of low ALI in LC. In addition, low ALI had obvious connection with inferior PFS/RFS (HR: 1.71, 95% CI: 1.35-2.07, *p* < 0.001) in LC patients.

**Conclusions:**

Low ALI before treatments indicates poor prognosis in LC patients. Serum ALI may serve as a promising predictive tumor marker of survival in LC sufferers.

## 1. Introduction

Lung cancer (LC) is one of the most common and aggressive malignancies worldwide [1, 2]. It is estimated that there are 234,030 new diagnosed cases of lung and bronchus cancer and 154,050 died from it in 2018 alone in the United States [1]. And LC ranked first in all of the cancer-related deaths in China [3, 4]. In the pathological type, non-small-cell lung cancer (NSCLC) and small-cell lung cancer (SCLC) account for 85% and 15%, respectively. Despite the development and improvements of diagnosis and treatments in LC, the prognosis of LC remains unsatisfactory, with a low long-term survival rate. It is of importance to find a novel index with an accurate predictive value in LC cases therefrom.

The advanced lung cancer inflammation index (ALI), as a newly reported inflammation-based prognostic score, is based on body mass index (BMI), serum albumin (ALB), and neutrophil to lymphocyte ratio (NLR). It was calculated as BMI × ALB/NLR [5]. The ALI was first evaluated as a powerful prognostic predictor in metastatic NSCLC [5], and subsequently, the usefulness of pretreatment ALI was evaluated as a prognostic marker in several types of malignancies, such as esophageal carcinoma and large B-cell lymphoma [6, 7]. In recent years, the role of ALI as a promising biomarker in LC attracted wide attention; more cohort studies worked on the relationship between pretreatment ALI and survival in LC patients [8–14]. However, the results in these published studies remain inconclusive [8–14], and no meta-analysis concerning the prognostic value of low ALI in LC patients is available so far. Therefore, in this study, we synthetically examined the correlation between pretreatment ALI and prognosis in LC via summarizing all currently available data. We hypothesized that low ALI could be a candidate predictor that is valuable for predicting survival and progression in LC.

## 2. Materials and Methods

### 2.1. Search Strategy and Study Selection

PubMed, Web of Science, and Embase were systematically searched for potential studies. The following keywords and terms were used: “advanced lung cancer inflammation index,” “ALI,” “lung cancer,” “lung tumor,” or “lung carcinoma.” The last update was Jan. 1, 2019, and no published language was restricted.

### 2.2. Inclusion and Exclusion Criteria

The inclusion criteria are as follows:

1. All recruited subjects were pathologically diagnosed with primary LC
2. The patients were divided into the low-ALI and high-ALI groups based on the pretreatment ALI levels
3. The HRs for the overall survival (OS), progression-free survival (PFS), or recurrence-free survival (RFS) were available

The exclusion criteria are as follows: reviews, abstracts, or posters or not involved in human lung cancer. And the latest study was included if there were overlapping data.

### 2.3. Data Extraction and Quality Assessment

Basic information of the included studies was extracted by two reviewers (Yi Zhang and Bo Chen), independently. The major features are listed in detail in Table 1. In addition, for the survival data, seven studies [5, 8–13] provided the HRs and 95% CIs in multivariate analysis for OS, and the HRs in multivariate analysis for PFS were reported in two studies [5, 14]. Only one study reported the HRs for RFS in univariate analysis [11]. If a study considered patients with high ALI as the reference, then, the data was converted to HR estimations considering cases with low ALI as a reference group to reflect the impact of low ALI on LC participants. Quality assessment was assessed using the method that was described in detail by Lin et al. [15].

### 2.4. Statistical Analysis

All statistical analyses were carried out using Stata/SE14.1 (Stata Corp LP, College Station, Texas, USA). The associations between low ALI and OS or PFS/RFS in lung cancer were expressed as the hazard ratios (HRs) with their corresponding 95% CIs. Heterogeneity across studies was evaluated by Cochran's *Q* test and Higgins' *I* ^2^ statistic. The fixed effect model was adopted for nonsignificant heterogeneity (*I* ^2^ < 50%, *p* > 0.1). Publication bias was assessed by the visible plot and Begg's test, and sensitivity analysis was performed for the measurement of the reliability of the combined results.

## 3. Results

**Figure 1:**
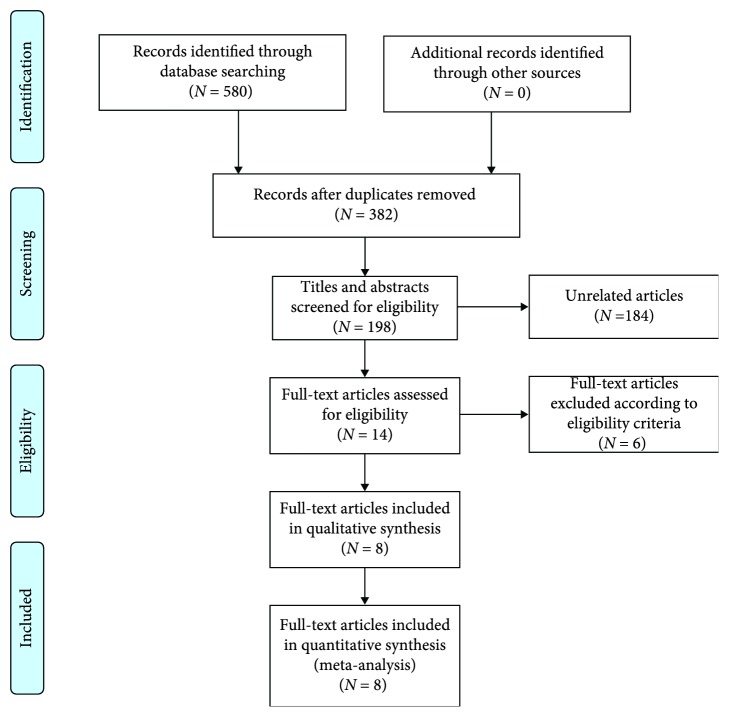
Flow diagram of included articles in the meta-analysis.

**Figure 2:**
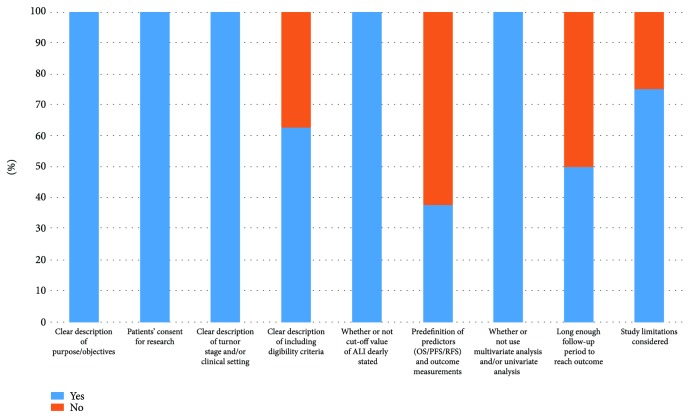
Quality assessment of 8 included studies.

The detailed selection procedure was listed (Figure 1). According to the abovementioned criteria, finally, a total of eight studies [5, 8–14] were considered to be eligible for this meta-analysis. There were altogether 1587 LC patients from eight retrospective studies with a mean sample size of 198.4. These studies were carried out in the USA (one study), China (one study), Korea (one study), France (one study), Japan (three studies), and Turkey (one study). Among them, seven studies reported OS, 2 studies covered PFS, and 1 study reported RFS. For the correlation between pretreatment ALI and OS, five studies worked on non-small-cell lung cancer (NSCLC), and two focused on small cell lung cancer (SCLC). The quality of the eight cohort studies was good with an average score of 7.25 (range 6-9; Figure 2, Table S1). The major characteristics of the recruited studies are summarized in Table 1.

### 3.1. ALI and OS

**Figure 3:**
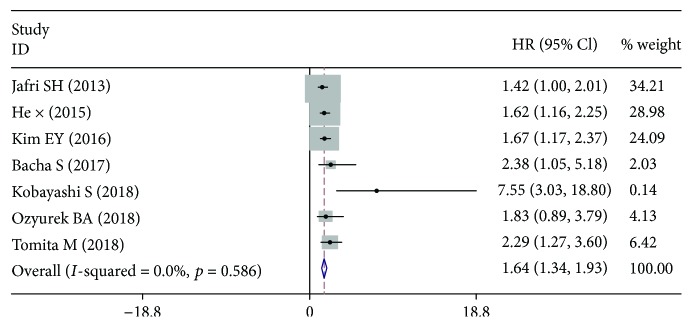
Forest plot for the correlation between low-ALI and OS in LC.

Seven cohort studies with a total of 1386 LC patients reported the HRs for the association between pretreatment ALI and OS. The pooled analysis found that pretreatment ALI was closely linked to the prognosis of OS, and the LC patients with low-ALI had a shorter survival time (HR: 1.64, 95% CI: 1.34-1.93, *p* < 0.001) (Figure 3 **)**.

**Figure 4:**
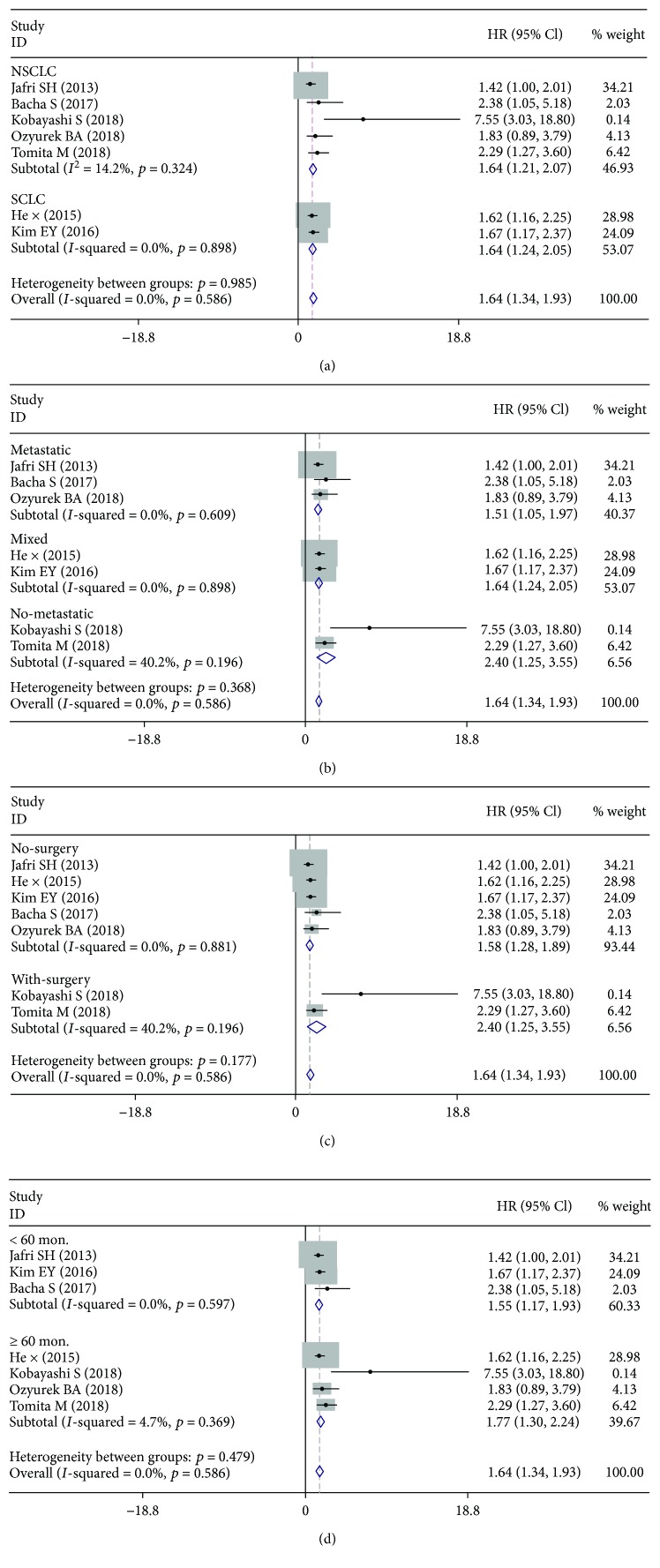
Forest plots for HRs of subgroup analyses for low ALI in LC.

The prognostic values of low ALI in lung cancer were further displayed in subgroup analyses (Figures 4(a)–4(d), Table 2). Notably, low ALI could act as an adverse prognostic factor of OS in NSCLC (HR: 1.64, 95% CI: 1.21-2.07, *p* < 0.001) and SCLC (HR: 1.64, 95% CI: 1.24-2.05, *p* < 0.001). And the clinical stages (metastatic vs. mixed vs. no metastatic), cut-off value (≥24.23 vs. <24.23), treatment methods (no surgery vs. with surgery), and the follow-up time (≥60 months vs. <60 months) all did not affect the significant predictive role of low-ALI in LC cases.

### 3.2. ALI and PFS/RFS

**Figure 5:**
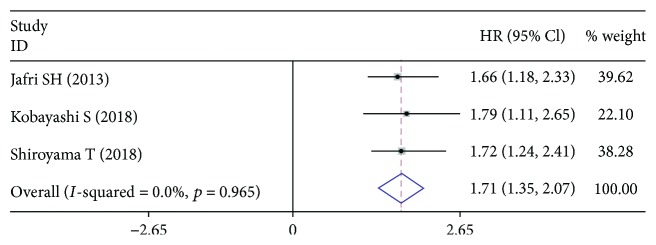
Forest plot for the correlation between low ALI and PFS/RFS in LC.

Three cohort studies with 540 LC subjects investigated the correlation between pretreatment ALI and PFS/RFS. The combined results showed pretreatment low ALI indicated worse PFS/RFS in LC (HR: 1.71, 95% CI: 1.35-2.07, *p* < 0.001), with no significant heterogeneity (*I* ^2^ = 0.0%, *p* = 0.965) (Figure 5 **)**.

### 3.3. Publication Bias

**Figure 6:**
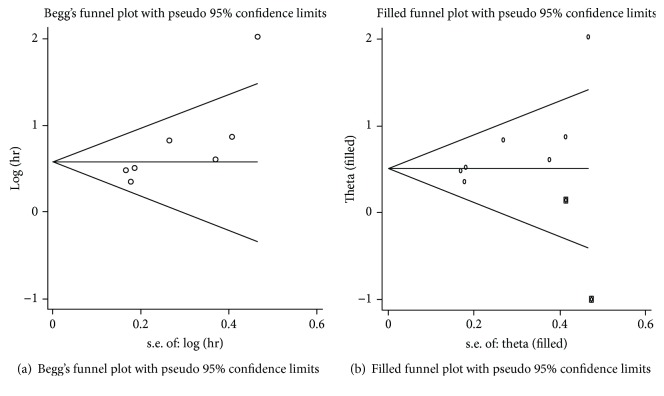
Publication bias assessment for OS: (a) Begg's funnel plot and (b) Filled funnel plot.

For the OS, the shape of Begg's funnel plot seems to be asymmetric (Pr > |*z*| = 0.035) (Figure 6(a)), the estimated pooled result was still significant (HR = 1.655, 95% CI: 1.402-1.952) after adjustment (Figure 6(b)) by the “trim-and-fill” method. For PFS/RFS, we did not conduct the publication bias assessment due to the small number of included studies.

### 3.4. Sensitivity Analysis

**Figure 7:**
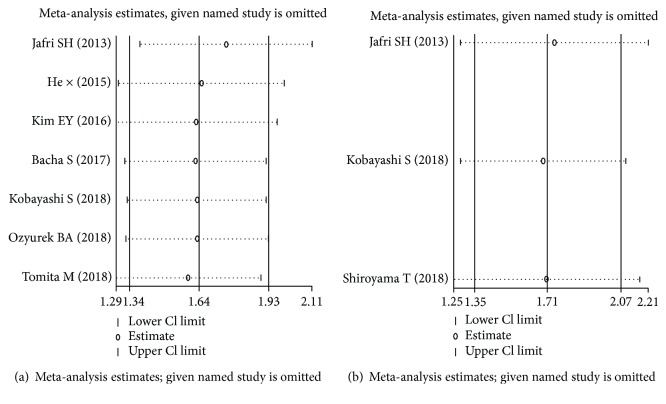
Sensitivity analysis for OS (a) and PFS/RFS (b).

The sensitivity analysis indicated the results of our analyses were relatively stable in this meta-analysis (Figure 7).

## 4. Discussion

In the current meta-analysis, a total of eight studies from six countries were collected for prognostic analysis. And to our knowledge, this is the first meta-analysis that focused on this field. Finally, there were altogether 1587 cases suffering from NSCLC or SCLC in this study. The combined results revealed a significantly shorter OS in LC cases with low ALI compared to those with high ALI, and low pretreatment ALI was also correlated with worse PFS/RFS in LC patients. Hence, these results suggested that a low ALI score was a promising indicator displaying negative prognostic values in LC patients.

The ALI is related to cancer survival and could be a prognostic indicator in cancer. However, the specific mechanism for the predictive value of this scoring system is uncertain. According to the definition, a low ALI based on a decreased BMI, a lower Alb, and/or a high NLR indicates poor prognosis and high systemic inflammation. BMI levels might represent the nutritional status of patients. Some studies reported that BMI could be a predictive factor of OS benefit in some tumors [16, 17]. In addition, numerous studies have clarified a close association of inflammation and cancer [18–21]; a series of inflammatory cytokines could reflect inflammatory response and participate in the tumor development and progression [21, 22]. Among them, serum albumin was demonstrated as an indicator in cancers; hypoalbuminemia was closely associated with poor prognosis in many tumors [23–27]. In lung cancer, the low level of Alb was defined as a valuable index of poor survival rate and worse response [28, 29]. Furthermore, neutrophils and lymphocytes were recognized as two important factors in carcinogenesis [30, 31], and high NLR could be used as an independent negative marker of predicting prognosis in lung cancer [32, 33] and other malignancies, such as breast, colorectal, and esophageal cancers [34–36].

Aside from the limited studies available, the present meta-analysis had several other limitations. Firstly, the included cohort data were of a retrospective design. Secondly, negative data were usually difficult to be published. Thirdly, there was a possibility of publication bias for OS. Fourthly, the prognostic role of low ALI for other secondary outcomes such as PFS/RFS in LC needed further validation. Finally, there are variations in several aspects, such as the ALI measurement methods and the cutoff values among these studies. Considering the limitations listed above, multicenter researches with a better-designed and larger sample are required to further validate the clinical value of ALI in LC.

In conclusion, from our analyses of eight cohort studies, we concluded that LC patients with a low ALI score have poor survival outcomes. LC patients with low-serum ALI had a shorter OS and worse PFS/RFS. Therefore, pretreatment ALI may act as a valuable candidate with a predictive power in LC.

## Figures and Tables

**Table 1:** Study characteristics of the eight cohort studies.

Study (year)	Region	Pathological type	Recruitment period	Total No. of cases	Gender (male/female)	Age (years)	End-point	Cut-off value	Selection method	Therapy	Stage	Follow-up (months)	Multivariate analysis
Jafri et al. (2013) [5]	USA	NSCLC	2000-2011	173	116/57	Median 57 (34-88)	OS, PFS	18	ROC curve	No surgery	Metastatic	<60	Yes
He et al. (2015) [8]	China	SCLC	2006-2011	365	310/55	Median 60 (22-82)	OS	19.5	Cutoff finder	No surgery	Mixed	≥60	Yes
Kim et al. (2016) [9]	Korea	SCLC	2010-2015	186	156/30	Mean 68.8 ± 9.4	OS	31.1	Using chi-squared test	No surgery	Mixed	<60	Yes
Bacha et al. (2017) [10]	France	NSCLC	2010-2012	41	41/0	Mean 56.3	OS	23.2	Median value	No surgery	Metastatic	<60	Yes
Kobayashi et al. (2018) [11]	Japan	NSCLC	2009-2014	166	74/92	NA	OS, RFS	22.2	ROC curve	With surgery	No metastatic	≥60	OS—yes; RFS—no
Ozyurek et al. (2018) [12]	Turkey	NSCLC	2006-2013	112	94/18	Mean 59.7 ± 9.9	OS	18	Median value	No surgery	Metastatic	≥60	Yes
Tomita et al. (2018) [13]	Japan	NSCLC	2008-2012	343	175/168	NA	OS	37.66	Cutoff finder	With surgery	No metastatic	≥60	Yes
Shiroyama et al. (2018) [14]	Japan	NSCLC	2015-2016	201	135/66	Median 68 (27-87)	PFS	18	Median value	No surgery	Metastatic	<60	Yes

All studies included were retrospective study. NSCLC: non-small-cell lung cancer; SCLC: small-cell lung cancer; OS: overall survival; RFS: recurrence-free survival; PFS: progression-free survival; ROC: receiver operating characteristic curve; NA: not available.

**Table 2:** Subgroup analyses of the relationship between ALI and OS in LC patients.

Subgroup factor	No. of cohort studies	Combined HR (95% CI)	*p* value	Heterogeneity	
				*I* ^2^ (%)	*P*_het_
*Pathological type*					
NSCLC	5	1.64 (1.21-2.07)	<0.001	14.2	0.329
SCLC	2	1.64 (1.24-2.05)	<0.001	0.0	0.898
*Clinical stage*					
Metastatic	3	1.51 (1.05-1.97)	<0.001	0.0	0.609
Nonmetastatic	2	1.64 (1.24-2.05)	<0.001	0.0	0.898
Mixed	2	2.40 (1.25-3.55)	<0.001	40.2	0.196
*Cut-off value*					
<24.23	5	1.57 (1.21-1.92)	<0.001	0.0	0.510
≥24.23	2	1.80 (1.27-2.33)	<0.001	0.0	0.351
*Treatment*					
No surgery	5	1.58 (1.28-1.89)	<0.001	0.0	0.881
With surgery	2	2.40 (1.25-3.55)	<0.001	40.2	0.196
*Follow-up*					
<60 m	3	1.55 (1.17-1.93)	<0.001	0.0	0.597
≥60 m	4	1.77 (1.30-2.24)	<0.001	4.7	0.369

## Data Availability

All the original data were available in the included published articles.

## Abbreviations

ALI: Advanced lung cancer inflammation index
LC: Lung cancer
NSCLC: Non-small-cell lung cancer
SCLC: Small cell lung cancer
BMI: Body mass index
ALB: Albumin
NLR: Neutrophil to lymphocyte ratio
HRs: Hazard ratios
OS: Overall survival
RFS: Recurrence-free survival
PFS: Progression-free survival
ROC: Receiver operating characteristic curve
NA: Not available.

## Supplementary Materials

Supplementary Materials Table S1: study quality of eight studies in the meta-analysis.
