# Supplementary material for: Prognostic Value of the Advanced Lung Cancer Inflammation Index in Patients with Lung Cancer: A Meta-Analysis
Source: Dis Markers. 2019 Jul 1;2019:2513026. doi: 10.1155/2019/2513026 (PMC6636448; doi:10.1155/2019/2513026)
Supplement: Supplementary Materials — Table S1: study quality of eight studies in the meta-analysis. [file 2513026.f1.docx]

Table S1. Study quality of eight studies in the meta-analysis.

| **Study [Reference]*** | **Country** | **Clear description of purpose/objectives** | **Patients' consent for research** | **Clear description of tumor stage and/or clinical setting** | **Clear description of including eligibility criteria** | **Whether or not cut-off value of ALI clearly stated** | **Predefinition of predictors (OS/PFS/RFS) and outcome measurements** | **Whether or not use multivariate analysis and/or univariate analysis** | **Long enough follow-up period to reach outcome** | **Study limitations considered** | **Quality score (0 − 9)** |
| --- | --- | --- | --- | --- | --- | --- | --- | --- | --- | --- | --- |
| Jafri SH (2013) | USA | yes | yes | yes | yes | yes | yes | yes | no | yes | 8 |
| He X (2015) | China | yes | yes | yes | yes | yes | yes | yes | yes | yes | 9 |
| Kim EY (2016) | Korea | yes | yes | yes | no | yes | no | yes | no | yes | 6 |
| Bacha S (2017) | France | yes | yes | yes | yes | yes | no | yes | no | no | 6 |
| Kobayashi S (2018) | Japan | yes | yes | yes | yes | yes | no | yes | yes | yes | 8 |
| Ozyurek BA (2018) | Turkey | yes | yes | yes | no | yes | no | yes | yes | no | 6 |
| Tomita M (2018) | Japan | yes | yes | yes | yes | yes | no | yes | yes | yes | 8 |
| Shiroyama T (2018) | Japan | yes | yes | yes | no | yes | yes | yes | no | yes | 7 |
